# Decoding Social Cues: A Scoping Review of AI-Supported Emotional Competencies and Their Impact on School-Aged Populations

**DOI:** 10.3390/jintelligence14090191

**Published:** 2026-09-01

**Authors:** Mabel Urrutia, Alonso Valderrama, Susana Araya, Luciano Silva, Francisco Ortiz, Cristian Medina, Pedro Salcedo, Pamela Guevara, Esteban J. Pino, Hipólito Marrero

**Affiliations:** 1 Facultad de Educación, Universidad de Concepción, Concepción 4030000, Chile; susaraya@udec.cl (S.A.); lsilva2017@udec.cl (L.S.); psalcedo@udec.cl (P.S.); 2 Facultad de Ingeniería, Universidad de Concepción, Concepción 4030000, Chile; alonso.valderrama@biomedica.udec.cl (A.V.); fortiz@ucsc.cl (F.O.); cmedina@doctoradoia.cl (C.M.); pguevara@udec.cl (P.G.); estebanpino@udec.cl (E.J.P.); 3 Facultad de Educación, Universidad Adventista de Chile, Chillán 3780000, Chile; 4 Facultad de Ciencias Económicas y Administrativas, Universidad Católica de la Santísima Concepción, Concepción 4090541, Chile; 5 Facultad de Psicología y Logopedia, Universidad de La Laguna, 38200 Laguna, Spain; hmarrero@ull.es; 6 Instituto Universitario de Neurociencia, Universidad de La Laguna, 38200 Laguna, Spain

**Keywords:** artificial intelligence, emotional education, virtual reality, automatic emotion recognition, scoping review, neuroscience

## Abstract

Developing emotional competencies has been identified as a key component of personal well-being, school coexistence, and significant learning during childhood and adolescence. The aim of this study was to map and categorise the quantitative scientific evidence available between 2019 and 2024 regarding the use of artificial intelligence technologies and algorithms applied to emotional education in school-age populations. This scoping review followed the PRISMA Extension for Scoping Reviews (PRISMA-ScR) and searched seven databases (Web of Science, Scopus, PubMed, PsycNet, ERIC, IEEE Xplore and the ACM Digital Library). The reviewed studies were published between the years 2019 and 2024. Eighteen studies met the inclusion criteria required for the final analysis. The results show that the most trained competence was machine-based emotional recognition using convolutional neural networks (CNNs). The goal of using them was to provide teacher support, specifically during initial or diagnostic assessments of students’ competencies. However, there remains a gap in its integration into the teaching and training of complex human skills, such as empathy. More longitudinal research is required, as well as more robust experimental designs that go beyond basic emotion models.

## 1. Introduction

Emotional competencies are a fundamental component of holistic education during childhood and adolescence, closely linked to pupils’ emotional development, interpersonal relationships, school life, well-being, and capacity to meet academic and social demands (Bisquerra Alzina & Pérez Escoda, 2007; Cipriano et al., 2023; UNESCO, 2024). Yet despite this widely acknowledged importance, developing and systematically assessing emotional competencies in school-age children continues to pose conceptual and methodological challenges. Recent work has shown that measurement instruments diverge in the constructs they assess, that their operational definitions remain inconsistent from one study to the next, and that the conditions under which school-based social–emotional learning programmes yield meaningful outcomes have yet to be fully established (Abrahams et al., 2019; Martinez-Yarza et al., 2023; Takizawa et al., 2023).

Over the past few years, artificial intelligence (AI) has emerged as a complementary means of analysing signals and indicators of emotional states in educational settings. AI-based systems draw on varied data—facial expressions, vocal characteristics, physiological signals, written language, and patterns of behavioural interaction—to detect, classify, or predict affective states (Khare et al., 2024; Wang et al., 2022). Such applications rest on machine learning and deep learning techniques, including convolutional and recurrent neural networks, and perform tasks such as facial and voice recognition, language analysis, multimodal processing, and affective monitoring, all within the broader field of affective computing (Ba & Hu, 2023; Vistorte et al., 2024; Yu et al., 2024).

In learning contexts, these technologies have been used to recognise facial expressions, estimate engagement, identify stress, analyse emotional language, and adapt technological interactions, and they have been delivered through social robots and intelligent tutoring systems. The wider educational literature has also examined virtual reality, chatbots, sentiment analysis, gamification, and wearable devices in relation to emotional learning (Sethi & Jain, 2024). Together, these applications point to possibilities for monitoring classrooms, generating alerts, adapting activities, or providing feedback to pupils, teachers, and other educational agents. The technical performance a system achieves in detecting or classifying affective signals does not, however, amount to evidence that pupils have acquired an emotional competency.

It is, therefore, necessary to distinguish among several things that are easily conflated: the computational functions of detection, classification, or prediction carried out by AI; the affective states, behaviours, or variables measured in individuals, such as joy, stress, or confidence, among others; the educational use made of these results through monitoring, alerts, feedback, or adaptation; and, finally, the evidence of learning or development of human emotional competencies, expressed as changes in the ability to recognise, express, and regulate emotions, and—in the case of empathy—to understand the emotions of others. Drawing this distinction is a conceptual and methodological priority for the present scoping review.

This review is grounded theoretically in Bisquerra Alzina’s model of emotional education (Bisquerra Alzina, 2009; Bisquerra Alzina & Mateo Andrés, 2019; Bisquerra Alzina & Pérez Escoda, 2007), which organises competencies into five blocks: emotional awareness, emotional regulation, emotional autonomy, life skills, and well-being. Its progressive, life-cycle orientation lends itself to analysing emotional development across the stages of schooling. Because the model was formulated to describe human capabilities rather than computational functions, however, applying it to AI-based studies calls for a careful separation between the conceptual correspondence of an emotional process and actual evidence of competency development.

Applying the model to AI-based research, therefore, required a process of operationalisation. Computational studies rarely evaluate broad domains, such as emotional autonomy or life and well-being competencies, as comprehensive educational constructs; instead, they process signals amenable to computational analysis—facial movements, vocal prosody, physiological responses, language, or behavioural patterns—in order to infer affective states, expressions, or processes (Ba & Hu, 2023; Khare et al., 2024; Vistorte et al., 2024; Wang et al., 2022; Yu et al., 2024).

For this reason, this review operationalised the framework through four analytical domains: emotional recognition, emotional expression, emotional regulation, and empathy. These domains allow us to classify, on the one hand, the affective processes addressed by computational systems and, on the other, the emotional skills assessed or supported in individuals, holding the two levels of analysis apart. The four domains were defined a priori and align with dimensions identified in the wider literature on emotional competence (Brackett et al., 2019; Grund & Holst, 2023).

At the human level, emotional recognition was tied to emotional awareness: the capacity to identify, differentiate, and understand one’s own emotional states and those of others (Bisquerra Alzina, 2003; Bisquerra Alzina & Pérez Escoda, 2007). For coding purposes, this domain comprised studies that analysed tasks involving the identification, discrimination, labelling, and basic understanding of one’s own and others’ emotional states. Recognition, so defined, does not exhaust the meaning of emotional awareness, which extends to broader processes of understanding and self-awareness that AI systems do not always evaluate.

Emotional expression was linked to emotional regulation, within which Bisquerra Alzina (2003) locates the ability to express emotions appropriately and to appreciate how such expression may affect others. For coding purposes, this domain covered studies in which AI analysed how emotions manifest through facial expressions, vocal characteristics, gestures, body posture, or language, as well as the intensity of and variation in these manifestations during an activity. Detecting or analysing an expression automatically is not, on its own, evidence that emotional expression has developed as a human competency.

Emotional regulation was associated with the ability to manage one’s own emotions and to draw on self-regulation strategies that appropriately modify, maintain, or modulate emotional responses (Bisquerra Alzina, 2003). For coding purposes, this domain took in studies that evaluated or supported self-regulation, coping, or the modification of emotional and behavioural responses during an activity. Automatically identifying negative emotions or affective shifts did not count as evidence of human emotional regulation.

Empathy, finally, was linked to the interpersonal and prosocial dimensions of social competence, particularly understanding others’ emotions, receptive communication, cooperation, and building respectful relationships (Bisquerra Alzina, 2003; Bisquerra Alzina & Mateo Andrés, 2019; Bisquerra Alzina & Pérez Escoda, 2007). For coding purposes, this domain comprised studies that explicitly evaluated or supported the understanding of other people’s emotional experiences, perspective taking, or empathetic and prosocial responses. Here, too, an AI system’s automatic identification of others’ emotions was not, on its own, treated as evidence of empathy as a human competency.

Recent reviews indicate that research has advanced both in automatic emotion recognition and in the exploration of educational applications. What remains limited is the integration among the computational performance of these systems, the pedagogical implementation of their outputs, and the evidence of pupils’ emotional learning (Yu et al., 2024). It is, consequently, far from clear whether these tools merely detect affective states or cues, whether their outputs guide educational decisions, or whether they furnish evidence that human emotional competencies have developed. This gap calls for a review that systematically separates the technical performance of the models, the educational functions implemented or proposed, and the evidence of change in emotional recognition, expression, regulation, and empathy.

The present review adopts a scoping review design, registered with INPLASY (INPLASY2025100064) and conducted in accordance with the PRISMA extension for scoping reviews (Tricco et al., 2018). Unlike reviews concerned with causal efficacy, scoping reviews aim to map the extent, nature, and characteristics of the available evidence, clarify key concepts, and identify research gaps (Peters et al., 2020).

The overall aim of this article is, therefore, to map and categorise the quantitative evidence published between 2019 and 2024 on the use of artificial intelligence technologies and algorithms in emotional education for school-age populations. To this end, this review was organised around three analytical dimensions. Dimension 1, demographic and contextual profiles, characterises the developmental stages, educational levels, and geographical distribution of the selected studies. Dimension 2, conceptual mapping and emotional evidence, identifies and classifies the social–emotional skills addressed—recognition, expression, regulation, and empathy—while distinguishing machine-based processes from learning at the pupil level. Dimension 3, technological architecture and educational function, classifies the data modalities and algorithmic techniques used and determines their analytical purpose and pedagogical use, whether to support teachers or to facilitate independent learning.

The result of this scoping review will help to report AI contributions to social–emotional development in school contexts and to provide guidelines for future research and educational practices.

## 2. Materials and Methods

This scoping review was conducted and reported in accordance with the PRISMA Extension for Scoping Reviews (PRISMA-ScR; Tricco et al., 2018). The review protocol was registered in INPLASY (registration number: INPLASY2025100064; https://inplasy.com/inplasy-2025-10-0064/ (accessed on 1 August 2026)). This framework supports transparent, reproducible, and methodologically rigorous reporting in evidence syntheses. This search was designed to identify studies on the integration of emerging technologies, such as artificial intelligence, virtual reality, and biofeedback, into educational contexts involving children and adolescents.

This article was organised around three analytical dimensions. The first characterised the demographic and contextual profiles of the evidence, considering stages of development, levels of education, geographical regions, the application setting, and the unit of analysis. The second dimension, conceptual mapping and emotional evidence, examined the domains of recognition, expression, regulation, and empathy, distinguishing between processes carried out by AI, affective variables measured in humans, and competences assessed or supported in students. The third dimension, technological architecture and educational function, characterised data modalities, algorithmic techniques, analytical purposes, and educational uses that have been implemented or proposed. Based on this structure, six research questions were formulated:

**Dimension 1: Demographic and contextual profiles**

- RQ1: How are AI-based studies and applications distributed according to stage of development, educational level, and the type of population or sample analysed?
- RQ2: What geographical and contextual patterns characterise the available evidence in terms of the distribution of studies across regions of the world?

**Dimension 2: Conceptual mapping and emotional evidence**

- RQ3: Which emotional domains—recognition, expression, regulation, and empathy-do AI-based studies with school-age populations address, and how do these relate to Bisquerra’s framework for emotional education?
- RQ4: What types of outcomes do the included studies report: machine-level emotional classification metrics, educational support outcomes, or student-level emotional learning outcomes?

**Dimension 3: Technological architecture and educational function**

- RQ5: Which data modalities and AI techniques predominate in the included studies, and for what analytical purposes—detection, classification, estimation, prediction, monitoring, adaptation, or intervention—are they used?
- RQ6: What educational functions do AI-based tools fulfil, and whom are they aimed at?

### 2.1. Search Strategy

To identify relevant studies, an initial search was carried out in three databases (Web of Science, Scopus, and PubMed) between June and September 2025. To complement this search, in June 2026, the database coverage was broadened with four additional databases (PsycNet, ERIC, ACM Digital Library, and IEEE Xplore). All searches were restricted to studies published between 2019 and 2024. This window was defined during the initial search, conducted between June and September 2025, when 2024 was the most recent year with complete database indexing. It was retained when coverage was later broadened, so that the original and complementary searches would remain comparable and free of partially indexed years. The figures reported in the following section correspond to this final, updated search across the seven databases. The search strategy combined four term blocks joined with the AND operator. The first block targeted artificial intelligence (artificial intelligence, machine learning, deep learning, AI techniques, and AI); the second targeted emotion-related concepts (emotion*, affective computing, simulation, and empathy); the third targeted technologies (serious games, technology, virtual reality, VR, biofeedback, neurofeedback, and avatar); and the fourth restricted the population to children and adolescents (child*, preschool, teenager*, adolescent*, and kid*).

The syntax used by the evaluators was as follows:

(ALL = (“artificial intelligence” OR “machine learning” OR “deep learning” OR “AI Techniques” OR “AI”) AND ALL = (“emotion*” OR “affective computing” OR “simulation” OR “empathy”) AND ALL = (“serious games” OR “technology” OR “virtual reality” OR “VR” OR “biofeedback” OR “neurofeedback” OR “avatar”) AND ALL = (“child*” OR “preschool” OR “teenager*” OR “adolescent*” OR “kid*”)).

In most cases, keywords were found by using the UNESCO Thesaurus. However, in some cases, it was not possible to find the exact term for the search, so the original terms were ultimately used. The search strings for the additional databases are in Table S1 in the Supplementary Materials.

### 2.2. Inclusion/Exclusion Criteria

Eligibility was structured following the population–concept–context (PCC) framework recommended by PRISMA-ScR: the population comprised typically developing children and adolescents aged 17 or younger; the concept was emotional competencies trained or supported through AI-based technologies; and the context was educational settings.

Studies were included if they met the following criteria:

1. Empirical study with a quantitative design. This criterion was applied to include studies reporting measurable results and to facilitate comparisons among them.
2. Date of publication between 2019 and 2024 in peer-reviewed scientific journals indexed in Web of Science (WoS), Scopus, PubMed, PsycNet, ERIC, IEEE Xplore or ACM Digital Library.
3. Participants were 17 years old or younger.
4. Employed AI-based technologies addressing emotional competencies, such as affective computing, emotion recognition (machine/deep learning), physiological regulation (neurofeedback and biofeedback), or immersive environments (VR, avatars, and serious games).
5. Studies must have been published in English.

The exclusion criteria were as follows:

- Research that was not related to education;
- Non-empirical studies (reviews, theoretical articles, and editorials);
- Studies on clinical or non-normotypical populations.

### 2.3. Search and Selection Process

The results of the search were processed using the Rayyan collaborative platform, a tool designed specifically to screen and organise references in systematic reviews. In total, 3731 records were identified through database searching: 790 from Web of Science, 188 from Scopus, 279 from PubMed, 212 from PsycNet, 9 from ERIC, 2253 from ACM Digital Library and 0 from IEEE Xplore. After removing 433 duplicate records, 3298 unique records underwent screening. Five reviewers with complementary disciplinary backgrounds (two postgraduate researchers in education and three in artificial intelligence) independently and blindly screened all records against the inclusion and exclusion criteria to minimise bias and ensure objectivity.

During the screening stage, the 3298 records were examined against the search keywords and eligibility criteria, and 3256 were excluded. The remaining 42 reports were sought for retrieval (all were successfully retrieved) and assessed for eligibility. After a comprehensive full-text reading, 25 reports were excluded, yielding 17 studies included through database searching. The excluded studies and the primary reason for exclusion for each study are presented in Supplementary Table S2.

In addition, a citation-searching process was conducted using the citation chaser tool, with the DOIs of the 17 database-identified studies as seed references. This procedure identified 1664 records, from which 1 report was sought, assessed for eligibility, and ultimately included. Consequently, the final review comprised 18 studies (17 identified through database searches and 1 through citation searches).

**Figure 1:**
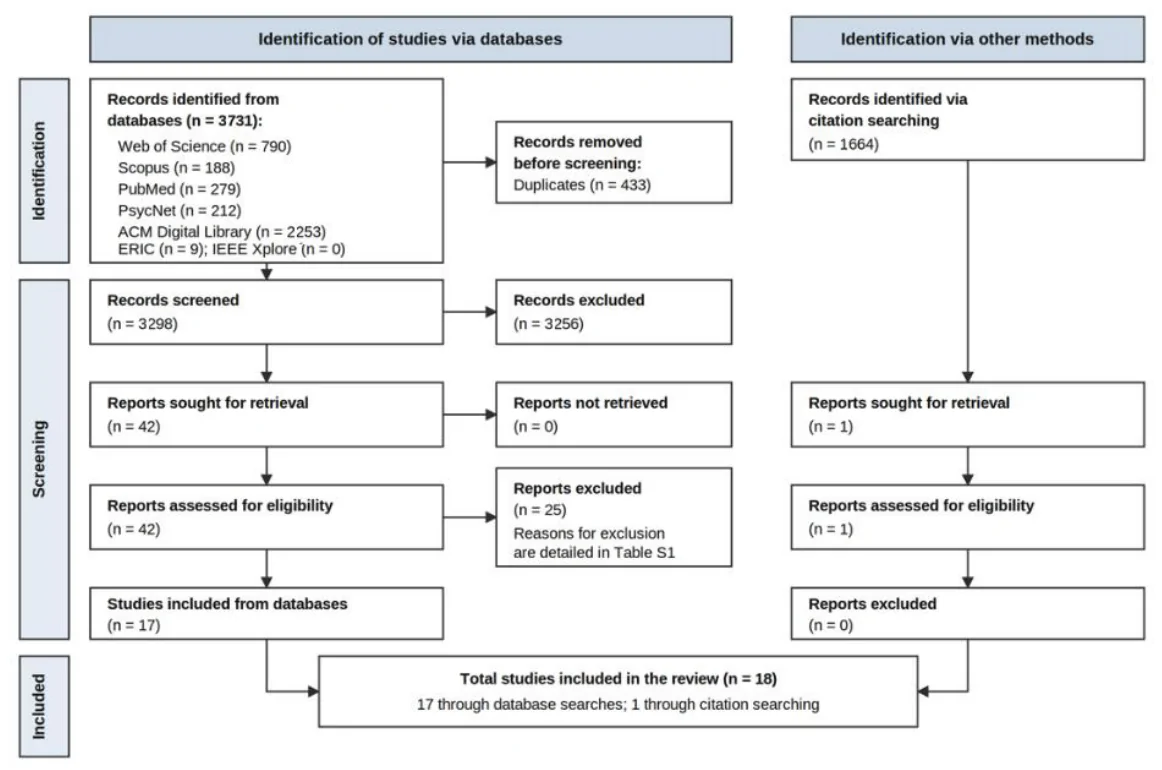
PRISMA-ScR flow diagram showing the identification, screening, eligibility, and inclusion stages of this review.

A PRISMA-ScR flow diagram was created to register the process and visually represent each of the search, sifting, and selection stages. The diagram details how many records were identified in each database, how many duplicates were found, and how many studies were excluded at each stage, as well as which were ultimately included in the analysis (see Figure 1).

### 2.4. Inter-Rater Reliability

To evaluate the consistency of the selection process, each reviewer’s pre-consensus decision was recovered from the Rayyan event log. Independent agreement at the title/abstract stage was high (percent agreement = 89%), with Fleiss’s kappa of 0.32 (95% CI: 0.27–0.37). This modest kappa likely reflects two factors: the low inclusion prevalence (~8.6%), which deflates kappa through the well-documented prevalence paradox, and the reviewers’ heterogeneous expertise, whose differing disciplinary perspectives can increase disagreement on borderline records. Consistent with the prevalence explanation, prevalence-robust coefficients indicated substantial-to-almost-perfect agreement (PABAK = 0.79; Gwet’s AC1 = 0.87). Discrepancies were resolved by consensus, and residual disagreements were adjudicated by an independent senior researcher to further ensure objectivity in the process.

### 2.5. Strategy for Data Synthesis

A previously piloted standardised data-charting form was used for the included articles. The purpose of this form was to organise, chart, and synthesise the relevant evidence. The following information was extracted: general information (author, country, and year); sample characteristics (size, population, etc.); experimental information (tools, educational setting, and reported outcomes); and findings (objectives and main results) (see Table 1).

### 2.6. Units of Analysis and Counting Rules

The study was the unit of analysis (*N* = 18), and every percentage in the results states its denominator. For mutually exclusive categories (the focus of training, the analytical purpose, the AI technique, the intended user, and the evaluation of effectiveness), percentages were computed over the 18 included studies and summed to 100%. Where a study could fall into more than one category, the coding unit was the assignment rather than the study; such percentages were not shares of studies, and the counts exceeded 18, namely 21 competence instances (three studies addressed two domains each), 24 competence-by-level counts (two studies whose samples spanned two stages were counted at both), and 26 intervention assignments (eight studies reported two types each). Emotional expression was retained as a coding category, although no included study addressed it.

## 3. Results

The results are organised around the three dimensions set out in the methodology. We begin with the first, demographic and contextual profiles. Table 1 summarises the main characteristics of the selected studies.

Table 1 shows all empirical studies included in this scoping review, as well as information regarding country of origin, type of population and sample size reported for each study. In the cases where the exact number of participants was not specified, the table shows “Unspecified”; otherwise, the corresponding analysis unit was included (i.e., drawings or recordings). Moreover, some articles did not report the exact chronological ages of the participants; therefore, the results are classified as those of children of unspecified age.

### 3.1. Geographical Distribution

The 18 included studies were conducted across nine countries, reflecting an international interest in the analysis of emotion in children and adolescents. China accounted for the largest share (seven of 18 studies (38.9%)), followed by India, Portugal, and South Korea (two studies each (11.1%)), and by Qatar, the United States, Spain, France, and Russia (one study each (5.6%)).

The studies were concentrated in Asia (12 of 18 studies (66.7%)) and Europe (five (27.8%)), with a single study from North America (5.6%), and reflected technological and cultural diversity in the tools and methods applied to these populations. All included studies were conducted in Northern-Hemisphere countries; no eligible study from South America, Africa, or Oceania was identified. This distribution reflects the retrieved evidence, not a geographic eligibility restriction, as no criterion limited the country or region of the studies.

### 3.2. Sample and Participants

The included studies showed high heterogeneity in their reported sample sizes, which ranged from six to 1328 in studies reporting human participants and from 81 to 291,651 in those reporting non-human units. This was partly due to the different units of analysis employed: 10 of the 18 studies (55.6%) reported human participants, six (33.3%) reported non-human units such as drawings, images, video frames, or videos, and two (11.1%) did not report a sample size. Consequently, these figures are not directly comparable across the reviewed literature.

Regarding the population, studies addressing children predominated (11 of 18 (61.1%)), followed by those involving adolescents (six (33.3%)) and preschoolers (three (16.7%)). These categories are not mutually exclusive, as two studies spanned more than one developmental stage (Kory-Westlund & Breazeal, 2019; Xie et al., 2022). Four studies (22.2%) worked with children but did not specify their chronological age. Consistent with the eligibility criteria, all included studies were conducted with typically developing populations.

Dimension 2, conceptual mapping and emotional evidence, turned to the specific emotional competencies most frequently addressed under Bisquerra’s model, and to the contribution AI made to them. Here, each application was classified as either machine-focused training or human-focused pedagogical development and examined across the different levels of schooling.

Across the 18 included studies, the four emotional domains were coded 21 times, as a study could address more than one competency; percentages are, therefore, reported both over the 21 coded competence instances and over the 18 included studies. Emotion recognition was by far the most frequently targeted competency, accounting for 17 of the 21 competence instances (81.0%) and present in 17 of the 18 studies (94.4%) (Ali et al., 2022; Calvo-Barajas et al., 2021; Han et al., 2021; Kalaiyarasi et al., 2024; Kim et al., 2021; Kory-Westlund & Breazeal, 2019; X. Li, 2022; S. Li et al., 2019; Lopez-Larrosa et al., 2023; Qayyum et al., 2023; Rathod et al., 2022; Savchenko et al., 2022; Veiga Simão et al., 2021; Xiao et al., 2024; Xu et al., 2024; Yan, 2022; Yun et al., 2020). Emotional regulation was considerably less common, with three instances (14.3%) in three studies (16.7%) (Han et al., 2021; Veiga Simão et al., 2021; Xie et al., 2022), and empathy with a single instance (4.8%) in one study (5.6%) (Xie et al., 2022). Notably, none of the reviewed studies targeted the expression of emotions (0%).

Since the competencies were not mutually exclusive, some studies overlapped across categories. Specifically, Han et al. (2021) and Veiga Simão et al. (2021) addressed both emotion recognition and regulation, whereas Xie et al. (2022) addressed both regulation and empathy. These three overlaps are what yielded 21 competence instances across 18 studies, the denominator used in Table 2.

The reviewed studies were categorised into two groups according to the focus of the emotional training: (a) studies using AI to train emotional competencies in machines and (b) studies aiming to develop them in human beings (see Table 2). The machine-focused group comprised 14 studies (77.8%), and the human-focused group comprised four studies (22.2%) (Calvo-Barajas et al., 2021; Han et al., 2021; Kory-Westlund & Breazeal, 2019; Veiga Simão et al., 2021). The two groups were mutually exclusive and, together, accounted for all 18 included studies.

As shown in Table 2, emotion recognition was the most frequently trained competence, accounting for 17 of the 21 competence instances (81.0%). It was trained in machines in 13 instances (61.9%; Ali et al., 2022; Kalaiyarasi et al., 2024; Kim et al., 2021; S. Li et al., 2019; X. Li, 2022; Lopez-Larrosa et al., 2023; Qayyum et al., 2023; Rathod et al., 2022; Savchenko et al., 2022; Xiao et al., 2024; Xu et al., 2024; Yan, 2022; Yun et al., 2020) and in humans in four instances (19.0%; Calvo-Barajas et al., 2021; Han et al., 2021; Kory-Westlund & Breazeal, 2019; Veiga Simão et al., 2021).

Emotional regulation accounted for three instances (14.3%): one in machines (4.8%; Xie et al., 2022) and two in humans (9.5%; Han et al., 2021; Veiga Simão et al., 2021). Empathy accounted for a single machine-focused instance (4.8%; Xie et al., 2022), with no human-focused instance. No study reported training of emotional expression. Table 3 reports how the included studies evaluated the effectiveness of this training.

Table 3 summarises how the included studies evaluated the effectiveness of emotional competency training, whether directed at AI systems or at pupils. Each study was assigned to one of two mutually exclusive categories. Most studies, 16 of 18 (88.9%), reported positive evidence of effectiveness, while two of 18 (11.1%) did not report adequate results (Ali et al., 2022; Yan, 2022).

Table 3 also records how far these claims were substantiated. Only four of 18 studies (22.2%) reported measures of effect size, statistical power, or practical significance; the remaining 14 of 18 (77.8%) reported none. Effectiveness in this corpus, therefore, rests largely on descriptive or self-reported assessment rather than on quantified estimates of magnitude.

**Figure 2:**
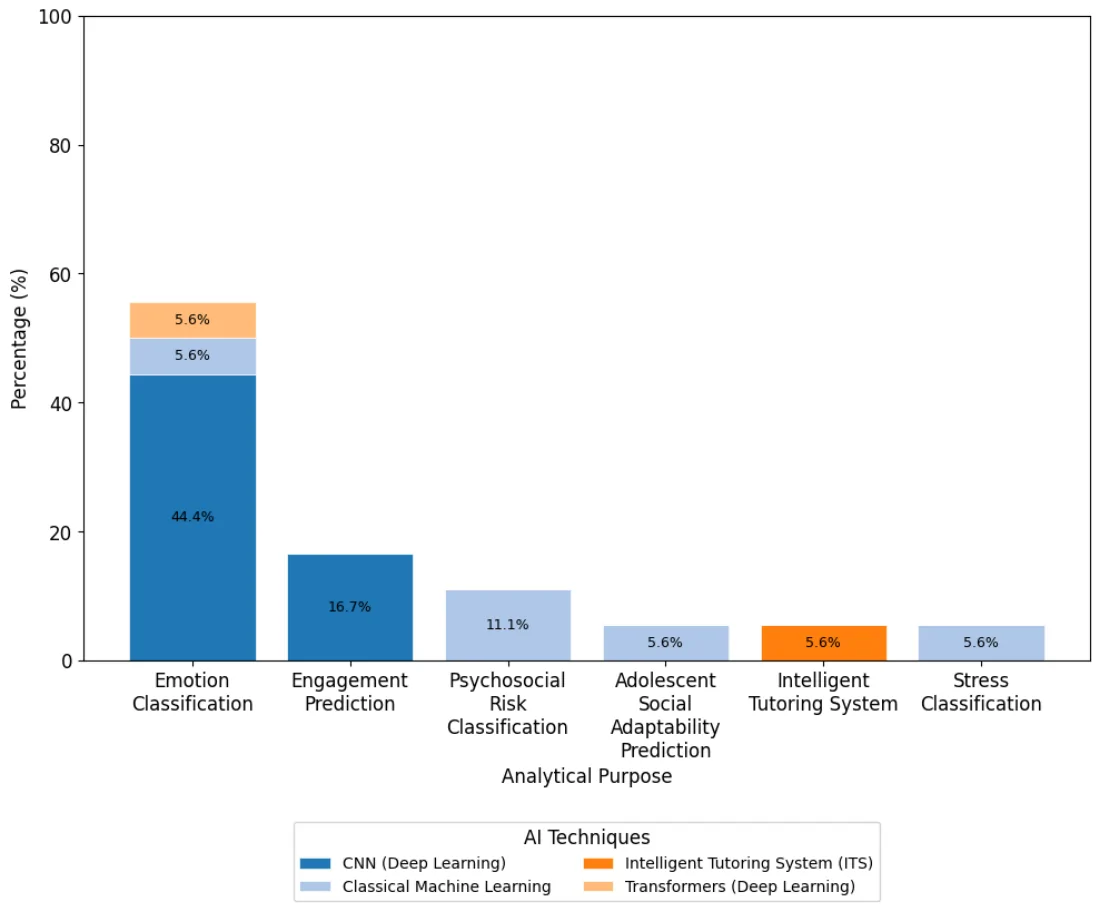
Frequency of the purpose of AI techniques. Note: Percentages were calculated over the 18 included studies. Each study appears once, classified by analytical purpose and AI technique; both classifications are mutually exclusive.

Figure 2 presents the AI techniques most frequently used to address emotional competencies, together with the analytical purposes these algorithms served in emotional training.

The reviewed studies were classified according to the analytical purpose for which AI algorithms were used (Figure 2). These categories are mutually exclusive and account for all 18 included studies. The most frequent purpose was emotion classification (10/18, 55.6%; Ali et al., 2022; Kalaiyarasi et al., 2024; Kory-Westlund & Breazeal, 2019; X. Li, 2022; Qayyum et al., 2023; Rathod et al., 2022; Veiga Simão et al., 2021; Xiao et al., 2024; Xu et al., 2024; Yan, 2022). This was followed by engagement prediction (3/18, 16.7%; Calvo-Barajas et al., 2021; Savchenko et al., 2022; Yun et al., 2020), stress classification (1/18, 5.6%; Kim et al., 2021), and psychosocial risk classification (2/18, 11.1%; S. Li et al., 2019; Lopez-Larrosa et al., 2023). Finally, one study each (1/18, 5.6%) addressed the prediction of adolescent social adaptability (Xie et al., 2022) and the implementation of an intelligent tutoring system (Han et al., 2021).

With regard to the techniques employed (Figure 2), convolutional neural networks (CNNs), including deep learning facial analysis architectures, were the most common (11/18, 61.1%; Ali et al., 2022; Calvo-Barajas et al., 2021; Kalaiyarasi et al., 2024; Kory-Westlund & Breazeal, 2019; X. Li, 2022; Rathod et al., 2022; Savchenko et al., 2022; Xiao et al., 2024; Xu et al., 2024; Yan, 2022; Yun et al., 2020). CNNs were primarily used to classify emotions from images and videos.

Classic machine learning models (tree-based algorithms, SVMs, and linear/NLP models) were used (5/18, 27.8%; Kim et al., 2021; S. Li et al., 2019; Lopez-Larrosa et al., 2023; Veiga Simão et al., 2021; Xie et al., 2022), applied to tabular data derived from facial, acoustic, physiological, or textual features. These models addressed stress detection (Kim et al., 2021), emotion detection (Veiga Simão et al., 2021), psychosocial risk classification (S. Li et al., 2019; Lopez-Larrosa et al., 2023), and the predictive analysis of adolescent social adaptability (Xie et al., 2022).

Transformer-based deep learning architectures were used in a single study (1/18, 5.6%; Qayyum et al., 2023) for emotion classification. Likewise, one study (1/18, 5.6%) implemented an intelligent tutoring system (Han et al., 2021), in which the emotional coding was carried out manually rather than through automated AI. These four technique categories are mutually exclusive and account for all 18 included studies.

**Figure 3:**
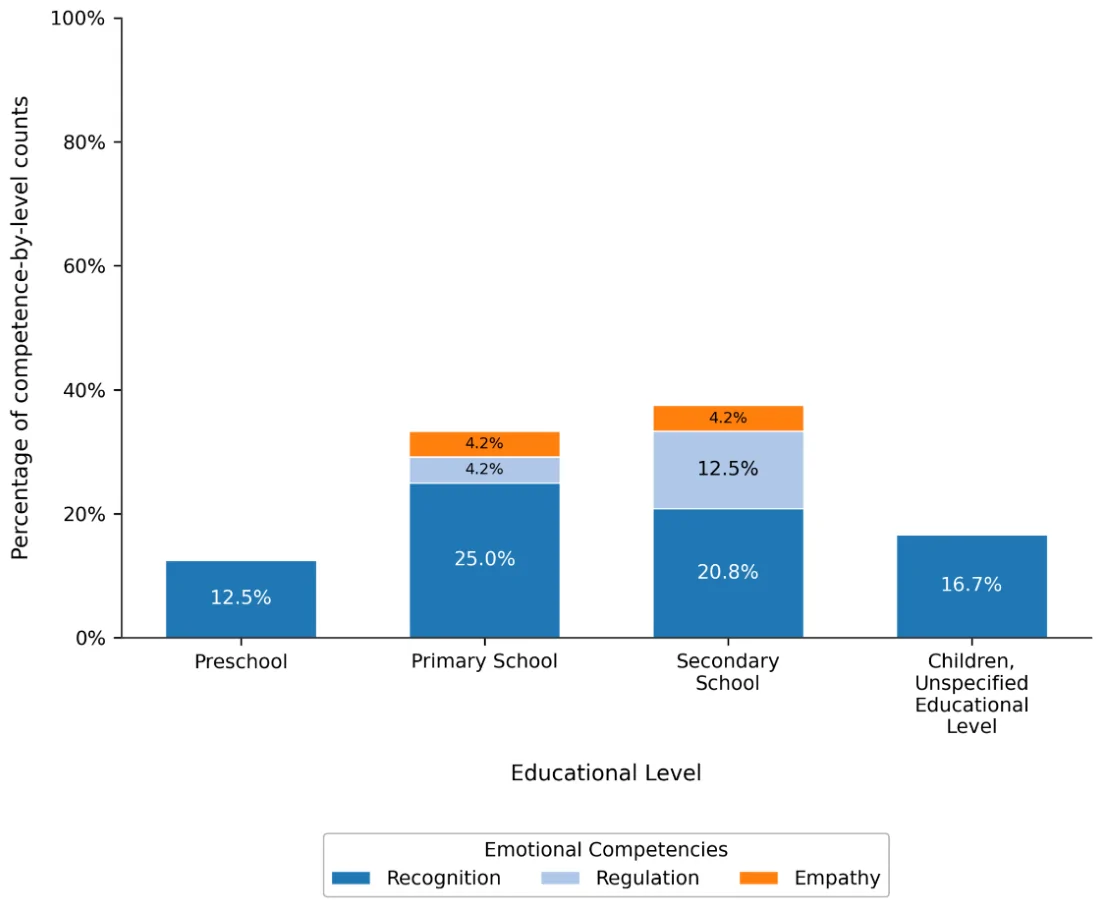
Emotional competencies according to the age range of children. Note: Percentages were calculated over the 24 competence-by-level counts coded across the 18 included studies. Two studies were counted at more than one developmental level, and three addressed more than one competency, so counts exceed the number of studies. Bar segments sum to 100% across the whole figure and are not proportions of studies within each level.

Figure 3 shows the age groups in which applications and tools for emotional competencies were most frequently used.

Figure 3 shows how the different emotional competencies were addressed with AI support across developmental levels. Of the 18 included studies, three involved preschool samples, seven primary, and six secondary, and four did not specify the age of the sample. Studies that worked with participants from more than one developmental stage, or that addressed more than one competency, were counted in more than one category, so the competence-by-level counts reported below total 24. The percentages reported below and in Figure 3 were calculated over those 24 counts.

At the preschool level, only emotion recognition was represented (3/24, 12.5%; Kory-Westlund & Breazeal, 2019; Xu et al., 2024; Yun et al., 2020).

At the primary level, emotion recognition was the most frequently addressed competency (6/24, 25.0%; Ali et al., 2022; Calvo-Barajas et al., 2021; Kory-Westlund & Breazeal, 2019; Qayyum et al., 2023; Rathod et al., 2022; Xiao et al., 2024), while emotional regulation and empathy were each addressed in a single study (1/24, 4.2% each; Xie et al., 2022).

The secondary level showed the largest representation. Emotion recognition remained the most common competency (5/24, 20.8%; Han et al., 2021; S. Li et al., 2019; X. Li, 2022; Lopez-Larrosa et al., 2023; Veiga Simão et al., 2021), followed by emotional regulation (3/24, 12.5%; Han et al., 2021; Veiga Simão et al., 2021; Xie et al., 2022) and empathy (1/24, 4.2%; Xie et al., 2022).

A further four studies addressed emotion recognition but were not assigned to a specific developmental level, as the age of the sample was not specified (4/24, 16.7%; Kalaiyarasi et al., 2024; Kim et al., 2021; Savchenko et al., 2022; Yan, 2022).

None of the reviewed studies addressed emotion expression (0%). Studies that spanned a broad age range were counted in more than one developmental level (Kory-Westlund & Breazeal, 2019 [preschool and primary]; Xie et al., 2022 [primary and secondary]).

**Figure 4:**
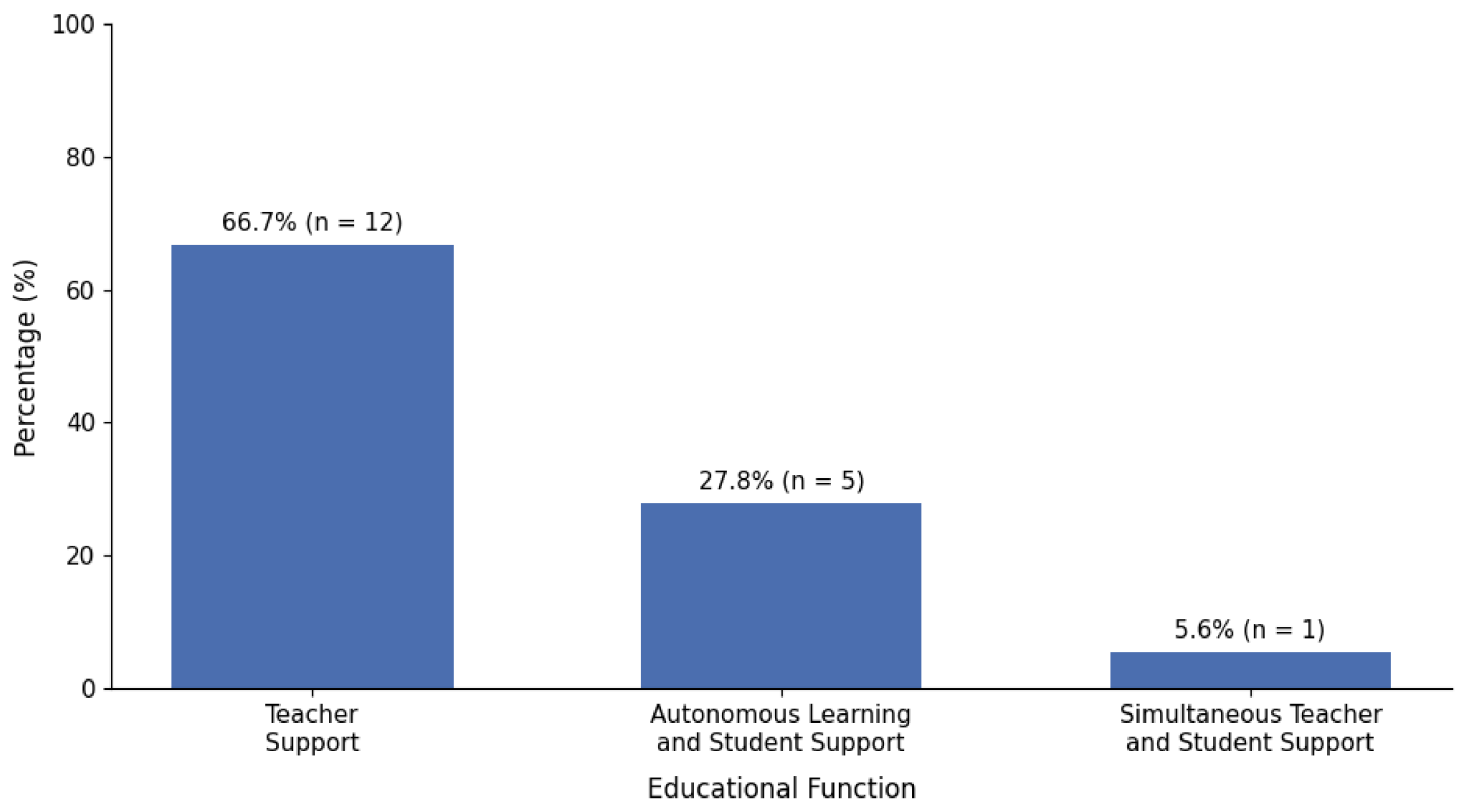
Distribution of AI-based tools according to their educational purpose. Note: Percentages were calculated over the 18 included studies. The three categories are mutually exclusive and sum to 100%.

Finally, Dimension 3, technological architecture and educational function, outlines the purposes for which emotional education software is used, as well as the specific aspects of teaching practice that AI tools support. To this end, the reviewed studies were classified into three mutually exclusive categories according to the intended user of the AI-based software, which, together, account for all 18 included studies (Figure 4).

Teacher support (12/18, 66.7%): These tools focus on providing feedback on the affective state of the classroom, easing differentiated instruction, and issuing early warnings about vulnerable students. Systems based on visual cues identify micro-expressions that correlate with comprehension or frustration, offering participation metrics in teacher dashboards (Savchenko et al., 2022; Yun et al., 2020). Multimodal approaches combining voice, text, and video provide real-time information on students’ affective states and early alerts about demotivation or risk (Kalaiyarasi et al., 2024; Kim et al., 2021; Xiao et al., 2024). Likewise, machine learning models applied to language and behavioural data support the early identification of vulnerable students (S. Li et al., 2019; Lopez-Larrosa et al., 2023).

Autonomous learning and student support (5/18, 27.8%): These tools are directed at the student to promote self-regulation, emotional literacy, and personalised learning. Social robots that adapt their intonation and gestures target participation and performance at early ages (Calvo-Barajas et al., 2021; Kory-Westlund & Breazeal, 2019), intelligent tutoring systems regulate students’ affect during self-directed learning (Han et al., 2021), and gamified mobile applications foster the recognition and management of affective states (Veiga Simão et al., 2021; Xie et al., 2022).

Simultaneous teacher and student support (1/18, 5.6%): This hybrid solution enables bidirectional interaction by combining automatic emotion recognition with explainable AI (XAI) models that expose the facial features underlying each inference (Rathod et al., 2022).

**Figure 5:**
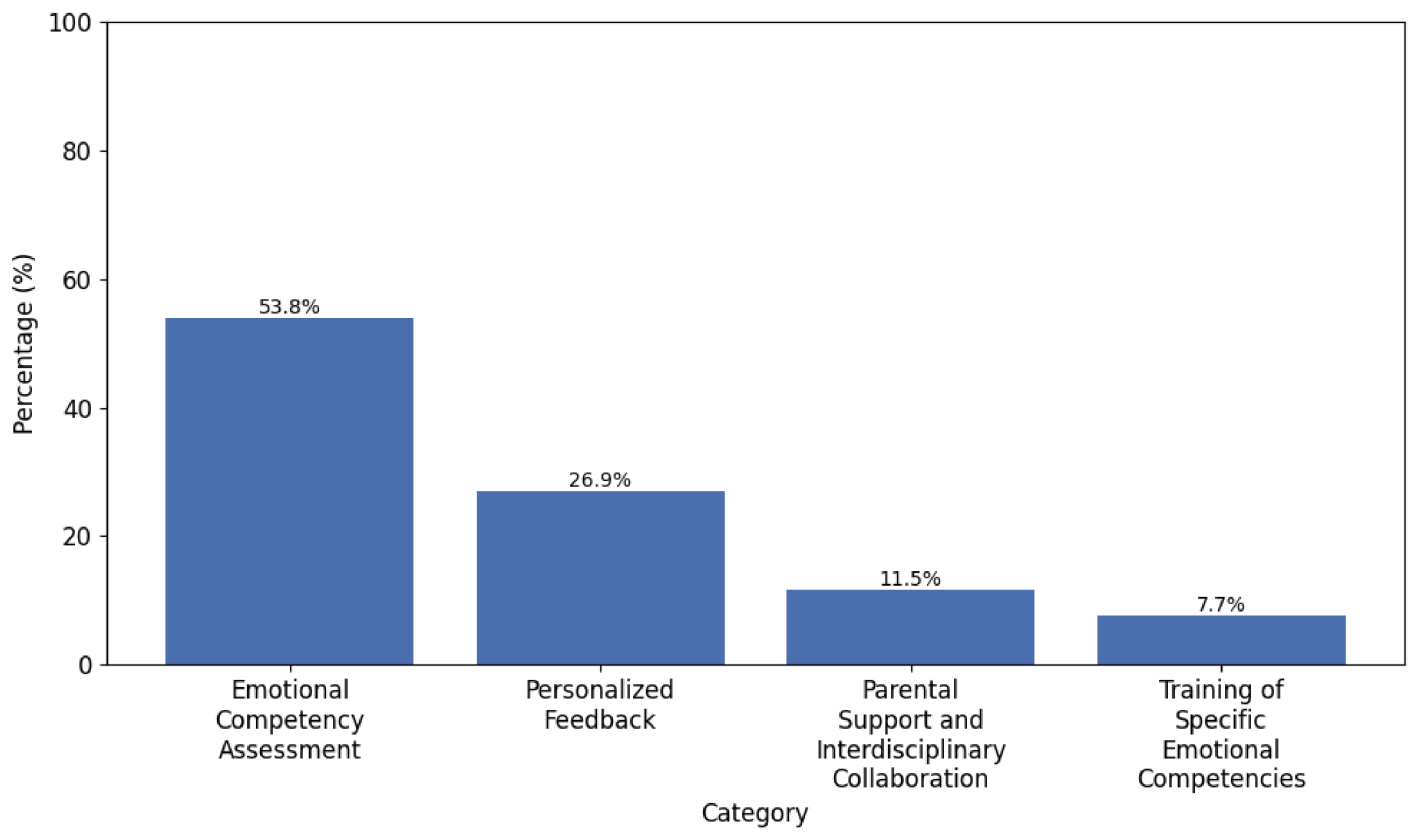
Frequency according to category of intervention on emotional competencies. Note: Percentages were calculated over the 26 intervention assignments coded across the 18 included studies. A study could fall into more than one category, so assignments exceed studies.

AI tools have eased the integration of innovative strategies into the assessment and strengthening of emotional competencies. Figure 5 shows the frequency of four non-exclusive categories of intervention used when addressing emotional competencies in educational contexts. Because a study could fall into more than one category, the 18 included studies yielded 26 category assignments, over which the percentages below and in Figure 5 were calculated.

Initial assessment of emotional competencies was by far the most frequent line of research: 14 assignments (14/26, 53.8%) used AI to identify students’ affective states. By using pre-trained (VGG-Face) CNNs, Yun et al. (2020) obtained precise real-time recognition of children’s engagement. Using an ordinal approach, Xu et al. (2024) combined a Siamese Network with intensity estimation to detect micro-changes in expression and improve the detection of emotional variation in children’s faces. Likewise, S. Li et al. (2019) applied natural language processing to adolescents’ diaries to estimate psychological resilience for early identification.

Training in specific emotional competencies was addressed in only two assignments (2/26, 7.7%), both of which focused on emotional regulation. Han et al. (2021) showed that a teachable-agent intelligent tutoring system (Betty’s Brain) promoted effective affect regulation strategies in students during autonomous learning. Veiga Simão et al. (2021) used a mobile application based on emotion and behavioural self-regulation strategies to help adolescents reduce aggressive online communication and foster prosocial behaviour.

Personalised feedback appeared in seven assignments (7/26, 26.9%). Calvo-Barajas et al. (2021) used Regulatory Focus Theory (RFT) to assess social robots that provide immediate feedback; this led to increases in children’s classroom trust, their perception of the robot’s competence, and their willingness to follow its recommendations, showing that immediate emotional feedback fosters interaction in educational environments. Similarly, Savchenko et al. (2022) delivered real-time engagement and emotion estimates to the teacher’s device, enabling lecturers to adjust their delivery during online lessons, while Rathod et al. (2022) added explainable AI (XAI) visualisations that expose the facial features supporting each inference, strengthening the user’s confidence in the feedback provided.

Parental support and interdisciplinary collaboration showed the lowest prevalence, appearing in three assignments (3/26, 11.5%). Kim et al. (2021) sent real-time stress alerts to the child’s guardians through a mobile application, enabling a rapid protective response, and Lopez-Larrosa et al. (2023) developed machine learning models that predict adolescents’ involvement in family conflict, information relevant for researchers and professionals providing clinical services. In all cases, the AI output was shared beyond the teacher–student dyad, favouring early detection of difficulties and effective coordination among the adults involved.

The reviewed studies show that when AI tools are applied to the development of emotional competencies, the results are mostly positive in terms of technical performance. However, it is important to differentiate between the technological effectiveness of tools (human–machine) and their actual impact on people’s training competencies (emotional education).

### 3.3. Effectiveness of Tools (Human–Machine)

Tools have demonstrated high technical performance; across the corpus, 88.9% of studies (16/18) reported effective results (Table 3). Rathod et al. (2022) reported up to 90.98% accuracy in detecting children’s emotions using deep learning models with explainable AI. Similarly, Kim et al. (2021) achieved 88.53% accuracy in identifying children’s stress states from voice and heart rate data, and Xu et al. (2024) reached 95.56% accuracy in detecting expression responses to emotional stimuli using an ordinal intensity estimation approach. These results support the tools’ technological capabilities, regardless of their impact on users’ emotional learning.

### 3.4. Effectiveness on Training People

Although tools operate at a technical level, their ability to develop users’ emotional competencies depends on their implementation. Of the 18 studies reviewed, only two (Han et al., 2021; Veiga Simão et al., 2021) were designed to actively train an emotional competency rather than merely detect it. Han et al. (2021) showed that a teachable-agent intelligent tutoring system (Betty’s Brain) promoted effective affect regulation strategies in students when integrated into a structured classroom activity, while Veiga Simão et al. (2021) found that a mobile application based on self-regulation strategies helped adolescents decrease aggressive online communication over time. In contrast, the remaining studies, such as those by Yun et al. (2020) and Xu et al. (2024), were limited to monitoring or classifying emotion. Consistently, only 22.2% of the studies (4/18) reported effect-size or practical significance measures (Table 3).

## 4. Discussion

The aim of this scoping review was to map and categorise the quantitative evidence published between 2019 and 2024 on the use of artificial intelligence technologies and algorithms in emotional education for school-age populations. In this article, we analysed the relationship between already used computer techniques and social–emotional competencies in three different dimensions: Dimension 1, demographic and contextual profiles; Dimension 2, conceptual mapping and emotional evidence; and Dimension 3, technological architecture and educational function. This section discusses the strengths and limitations of each dimension. The purpose of this analysis was to determine AI contributions to social–emotional development in school contexts.

The results show that automatic emotion recognition (AER) has become relevant in education, thanks to recent progress in AI and automated learning. Different studies have documented their potential in improving the process of both teaching and learning by allowing real-time monitoring of the emotional and cognitive commitment of students (see Calvo-Barajas et al., 2021).

Interventions developed with the support of algorithms tend to focus on skills represented through patterns, such as emotional recognition. This can be explained by their ability to process large amounts of data arising out of facial cues (Han et al., 2021; Kalaiyarasi et al., 2024; Kory-Westlund & Breazeal, 2019; Savchenko et al., 2022; Xiao et al., 2024; Xu et al., 2024), voice signals (Kalaiyarasi et al., 2024; S. Li et al., 2019; Qayyum et al., 2023) and text analysis (Kalaiyarasi et al., 2024; S. Li et al., 2019). Conversely, human interventions tend to work on more complex emotional competencies, such as emotional regulation and empathy. These demand comprehension of the context and relational adaptation (Veiga Simão et al., 2021; Xie et al., 2022).

In terms of emotional regulation, Han et al. (2021) showed that AI-based tools contribute to promoting effective affect regulation strategies in students through cognitive and metacognitive scaffolding. The authors’ study evaluated a teachable-agent intelligent tutoring system that uses virtual characters and dialogue interaction to modulate emotion in educational environments by providing timely feedback and cause-and-effect problem-solving tasks.

In terms of empathy in AI-based interventions, they are underrepresented compared with human interventions, as they are essentially intersubjective and reflexive. Xie et al. (2022) pointed out that promoting empathy requires dynamic interactions that adjust to the emotional context. Consequently, they remain effective in human contexts where individuals can adaptively respond to complex signals.

The analysed corpus showed an imbalance in terms of training of emotional competencies, with notable differences favouring emotion recognition, particularly in machines rather than humans. According to our results, AER primarily focuses on enhancing teacher sensitivity and fostering student self-regulation. The development of hybrid systems is a future research goal and requires participatory methodologies that involve all stakeholders to ensure their relevance and sustainability.

With regard to AI algorithms, CNNs are the most widely used tool, as they can extract features from highly dimensional data, such as images, video and biometric signals. Our results show that there is a direct relation between these algorithms and techniques currently being used, as well as with available data. This is why it is the most commonly used technique for emotion classification, as the most commonly available types of data are video, voice and text. Although we found studies involving biometric signals, there is still a need for a greater corpus of electrophysiological or electrodynamic signals to apply different algorithms, such as tree-based algorithms, SVMs, and linear models. Using such networks enables real-time facial analysis, which, in turn, enables the creation of immediate social–emotional diagnostic tools, helping teachers adjust their teaching strategies. However, the current approach focuses more closely on machines rather than humans. Conversely, there is low representation of Q-learning algorithms, which reflects a lack of inclusion of different AI tools in teaching social interaction between students and educators.

Regarding the educational purpose of classroom AI tools, the results show that they primarily focus on teacher support rather than autonomous student learning, driven heavily by tools designed to assess students’ affective and motivational states. As highlighted in the accompanying graph, emotional competency assessment constitutes the largest share of these interventions, at 53.8%. Only a small portion of the reviewed software fosters direct teacher–student interactions, a limitation closely related to technical architectures that prioritise machine-centred classification over human development.

Conversely, social robots contribute increasingly to hybrid solutions by delivering immediate emotional feedback, which falls under the 26.9% of applications providing personalised feedback. While machine learning models are widely used to predict emotional states and enable customised learning, parental support remains poorly represented, at just 11.5%, underscoring a persistent challenge in social interactions between schools and the wider educational community.

The findings of this scoping review demonstrate that AI tools achieve high technical effectiveness, particularly for emotion classification, which constitutes the predominant analytical purpose, at 55.6% of the reviewed studies. Across the corpus, algorithms consistently yield strong classification and precision metrics when analysing voice cues, children’s drawings, and multimodal stimuli. For human interaction, virtual-reality-based tools adapted best to empathy training and social interaction. However, a widespread lack of statistical power across the corpus limits the generalisability of these strong technical findings. Consequently, despite these promising preliminary metrics, the true scope of these technologies within formal educational settings remains undetermined.

That being said, most of the reviewed studies from recent years (e.g., Banos et al., 2024; Jaliaawala & Khan, 2020; Landowska et al., 2022) focused on clinical settings or therapeutic interventions, specifically autism spectrum disorder (ASD). The fundamental contribution of current studies lies in analysing the impact of AI on teacher support and social dynamics of the classroom for an extended age range of typically developing individuals.

A common aspect between our results and similar reviews lies in the predominance of emotion recognition training in studies over other emotional competencies, such as regulation. In this regard, both Landowska et al. (2022) and Banos et al. (2024) also identified that most of the automated systems are limited to the perception stage (identifying basic facial expressions, like joy or sadness).

Meanwhile, the low representation of AI tools aimed at training complex social competencies like empathy is consistent with Davis’s (2024) conclusions. The author holds that AI shows a “weak intelligence” when addressing cognitive processes underlying real human empathy. In line with our findings, it has been suggested that virtual environments are efficient tools for the work of dimensions preceding social competence. In this line, Moore et al. (2005) highlighted the usefulness of collaborative virtual environments for facial expression recognition and when addressing deficiencies in the Theory of Mind. Such skills, along with the ability to infer third parties’ emotions (Kandalaft et al., 2013), show how efficient immersive technologies are when training complex social cognition. Likewise, they allow for the projection of virtual environments as a resource with the potential to overcome current limitations seen in the analysed AI techniques.

In general terms, our findings show that CNNs are positioning themselves as some of the most commonly used AI techniques among our sample. However, despite the rise of deep learning, (Banos et al., 2024) found that SVM techniques are predominantly present in the literature. This observation is consistent with the results of this study, where SVMs were found to be one of the most frequently used techniques after tree-based algorithms.

### 4.1. Theoretical Implications

The results of this research reveal that one of the most frequently applied emotional competencies is emotion recognition; however, most of the findings focused on machine-based emotion training. Consequently, it is necessary to distinguish between the computational functions performed by machines (detection, classification, and prediction) and the internal psychological constructs and affective behaviours measured in humans. This avoids the fallacy of assuming that the technical performance or accuracy of an algorithm is, in and of itself, equivalent to the development of a human emotional competency. Notwithstanding the above, it is interesting to discuss, as Yu et al. (2024) propose in their systematic review, that emotion recognition has been studied mainly from a computer science perspective, with an emphasis on the technical development of AI, whilst its pedagogical value and educational applications have received less attention. Therefore, it is necessary to broaden the educational perspective to include elements of affective computing that could change the current view of the development of emotional competencies in the classroom.

Based on the objective of identifying age profiles for the implementation of AI in different educational contexts, we found a developmental pattern in the design of emotional education tools. They tended to train competencies only partially by prioritising those that align with the cognitive or social development features of each developmental stage. In this regard, there was a clear trend to prioritise emotion recognition in early and middle childhood (4–12 years old), while the focus was less during adolescence (13–18 years old). There were fewer studies analysing this stage. Also, they focused on more complex skills, such as emotional regulation. We also found a lack of studies explicitly working on emotional expression in any age range. Notwithstanding the foregoing, the emotional competence educational model (Bisquerra Alzina, 2009; Bisquerra Alzina & Mateo Andrés, 2019; Bisquerra Alzina & Pérez Escoda, 2007) holds that it is necessary to train all emotional competencies, which are operationalised in four central axes: recognition, expression, emotional regulation and empathy. They must be developed gradually at each stage of human development to achieve comprehensive emotional development.

Bisquerra Alzina ’s assumptions are linked to the guidelines adopted by UNESCO, which define social–emotional learning as a progressive and continuous process throughout the lifespan that encompasses all skills from the outset, with their level of complexity increasing only over time (UNESCO, 2024). Consequently, the fragmented focus on the development of emotional competencies in AI models is not in line with the idea that awareness, expression, and regulation are interconnected processes that must mature in parallel (UNESCO, 2024). Emotion recognition, for example, is a competency that stems from emotional awareness, a more complex construct that develops with age. Thus, while children can make progress in recognising emotions, over time, they learn to identify contradictions between what is expressed and what is felt, ultimately leading to the development of moral judgment (UNESCO, 2024). Furthermore, the expression of emotions is related to assertive communication, which is essential for community life. The lack of development in this competency highlights the need to develop intelligent systems capable of not only detecting but also interacting with and modelling the user’s responses, ensuring that AI support is more than just a passive or software-automated phenomenon (Picard, 1997).

### 4.2. Practical Implications

The findings of this scoping review establish that the primary pedagogical function of AI is to support teaching, particularly during the classroom assessment phase. Consequently, educational institutions could implement systems based on CNNs to conduct automatic diagnostic assessments of student engagement. Such tools would facilitate the management of individual differences and assist in planning class activities within a more inclusive learning environment.

Moreover, educational software capable of processing multimodal signals can generate early warnings regarding vulnerable, demotivated, or at-risk students, enabling timely and personalised interventions outside the classroom. Additionally, interfaces and user interactions should be designed to align with students’ developmental stages and cultural differences, thereby addressing emotional competencies throughout their academic journey.

### 4.3. Limitations and Future Research

One limitation of this study is that all identified evidence came solely from the Northern Hemisphere (primarily China, followed collectively by the European countries, with the remaining evidence distributed across India, South Korea, Qatar, Russia, and the United States), leaving a gap in research representation from South America, Africa, and Oceania. This geographical imbalance likely stems from language and indexing restrictions within academic databases, which only included English-language articles published in highly indexed journals while excluding potentially relevant qualitative studies from other regions.

Meanwhile, the studies included in the sample exhibited certain limitations. The use of multimodal data (facial cues, voice input, texts and clinical surveys) tended to be predominant in most of the reviewed studies (Banos et al., 2024; Jaliaawala & Khan, 2020; Landowska et al., 2022). Nevertheless, and in line with Banos et al. (2024), there is still a need for more extensive use of physiological sensors (skin conductivity and heart rate) to optimise emotional detection algorithms. Likewise, a critical limitation detected by our study was the lack of representation of data coming from neuroimaging and signal analysis (fMRI, EEG, and fNIRS). This is a methodological gap that, according to Banos et al. (2024), restricts the understanding of deep biological correlates in social–emotional learning. Regarding future research, neuroscientific studies are expected to increase, driven by recent advances in AI algorithms.

Another limitation identified by this scoping review is the tendency to utilise AI tools primarily as teacher support for the initial assessment of competencies. Consequently, a gap exists in the literature regarding hybrid systems that facilitate direct interactions between teachers and students during emotional competence training. In contrast, clinical research, such as that conducted by Cunningham et al. (2023), is already examining the complexities of the therapeutic relationship to enhance professional empathy. Such models could be successfully transferred to the educational context to foster more meaningful classroom dynamics.

In the same vein, while many studies have focused on the accuracy of AER via AI, limited research exists regarding how interaction with AI systems enhances users’ emotional skills, such as empathy, regulation, or general emotional wellbeing. In this context, the meta-analysis by X. Li et al. (2025) on VR for social–emotional learning (SEL) represents a fundamental step forward, demonstrating that technology can facilitate substantial improvements, particularly regarding complex social skills.

Likewise, there is a lack of methodological rigour that compromises the internal validity of the reviewed studies, most of which had limited samples or lacked control groups. However, AI can help solve this lack of statistical power for future research by using nig Data extracted from brain-imaging techniques. While these limitations affect mainly the quantitative corpus, the literature also reports a lack of qualitative or mixed approaches. On this subject, Jaliaawala and Khan (2020) highlight that the lack of standardised experimental designs, in particular, for technological interventions, compromises the validity of results. Ultimately, this lack of methodological rigorousness prevents a deep understanding of underlying cognitive mechanisms of students during emotional learning, creating a gap that the current literature fails to cover.

Finally, although the age profile of the sample was broad (as it included early childhood to adolescence), the results showed fragmented development of emotional competencies. In particular, there was a predominance of emotional recognition during childhood, while emotional regulation seemed to become more relevant during adolescence. As a result, more longitudinal studies are required in order to assess the gradual and progressive transfer of these skills in the long term, as proposed in Bisquerra Alzina’ s emotional competence model (Bisquerra Alzina & Mateo Andrés, 2019; Bisquerra Alzina & Pérez Escoda, 2007; Bisquerra Alzina, 2009). This approach implies addressing the complexity of emotions beyond all six basic universal emotions or simple dimensional models.

## 5. Conclusions

This scoping review shows that the field of artificial intelligence applied to emotional education is technically mature, but in a budding integration stage in teaching practice. Our results demonstrate that, while there is a high algorithmic precision for the classification of emotions (particularly through the use of CNNs and SVMs), the ability is mainly focused on the recognition of emotions by machines, relegating regulation and complex social interaction to the background.

A key contribution of this study when compared with other reviews is its focus on educational contexts and a wide age range of the school population. In contrast, many other reviews took a clinical approach. This study adhered rigorously to the PRISMA method, focusing on a recent period that includes the rise of generative AI and deep learning in the development of emotional competencies among different parties within the educational community.

Meanwhile, the study of emotional competencies was addressed from a comprehensive point of view by analysing its use in classroom dynamics. Our findings emphasise the role of AI as a teacher support tool during a diagnostic evaluation. Despite this, they reflect a lack of hybrid systems aimed at strengthening social interactions between students and teachers. In this framework, empathy showed the lowest prevalence among the competencies identified in the literature. However, breakthroughs in VR and robotics suggest a promising outlook for developing such a complex social skill in the near future.

This scoping review reveals several key limitations: a Northern Hemisphere geographic bias stemming from linguistic and indexing constraints, a notable scarcity of research integrating physiological and neuroimaging data, and a general lack of methodological rigour across the reviewed studies. Functionally, current AI tools are predominantly limited to teacher support, overlooking their potential to foster direct, interactive student–teacher engagement. Similarly, the lack of longitudinal studies hinders assessment of the impact and consolidation of such competencies over time.

Finally, we hold that the future of AI-assisted emotional education depends on going beyond basic universal models and simple dimensional approaches. It is necessary to integrate comprehensive theoretical frameworks like Bisquerra’s (Bisquerra Alzina & Mateo Andrés, 2019; Bisquerra Alzina & Pérez Escoda, 2007; Bisquerra Alzina, 2009), as well as promoting longitudinal research that accounts for the real transfer of these skills throughout student development. Only by aligning the potential of deep learning with the complex social–emotional needs of typically developing populations will it be possible to design tools that not only identify emotions but also strengthen students’ autonomy and wellbeing in their social environment.

## Figures and Tables

**Table 1:** Characteristics of studies included in this scoping review.

ID	Authors	Country	Population	Sample
1	Ali et al. (2022)	Qatar	Children	623 children’s drawings
2	Calvo-Barajas et al. (2021)	Portugal	Children	69
3	Han et al. (2021)	China	Adolescents	72
4	Kory-Westlund and Breazeal (2019)	USA	Preschool/children	86
5	Kalaiyarasi et al. (2024)	India	Children of unspecified age	Unspecified
6	Kim et al. (2021)	South Korea	Children of unspecified age	6
7	X. Li (2022)	China	Adolescents	776 images
8	S. Li et al. (2019)	China	Adolescents	82 Participants
9	Xiao et al. (2024)	China	Children	3598 labelled instances
10	Lopez-Larrosa et al. (2023)	Spain	Adolescents	247
11	Qayyum et al. (2023)	France	Children	26,000 video frames
12	Rathod et al. (2022)	India	Children	81 videos
13	Savchenko et al. (2022)	Russia	Children of unspecified age	291,651 images
14	Veiga Simão et al. (2021)	Portugal	Adolescents	218
15	Xie et al. (2022)	China	Children, adolescents	1328
16	Xu et al. (2024)	China	Preschool	45
17	Yan (2022)	China	Children of unspecified age	Unspecified
18	Yun et al. (2020)	South Korea	Preschool	30

**Table 2:** Trained emotional competence by focus.

Competence	Focus	Instances (*n*)	% of 21 Instances
Recognition	In machines	13	61.9
	In humans	4	19.0
Regulation	In machines	1	4.8
	In humans	2	9.5
Empathy	In machines	1	4.8
	In humans	0	0.0
Expression	In machines	0	0.0
	In humans	0	0.0
**Total**		**21**	**100.0**

Note. Percentages were calculated over the 21 competence instances coded across the 18 included studies. A study contributed one instance for each competence it addressed; three studies addressed two competences each (Han et al., 2021; Veiga Simão et al., 2021; Xie et al., 2022), which is why instances exceed studies. Bold indicates the total row.

**Table 3:** Evaluation of training effectiveness across the 18 included studies.

Category	Studies (*n*)	% of 18 Studies
**Overall assessment of effectiveness**		
Effective	16	88.9
Not effective	2	11.1
**Substantiation of effectiveness claims**		
Effect-size or practical significance measures reported	4	22.2
No such measures reported	14	77.8

Note: Percentages were calculated over the 18 included studies. Each block is mutually exclusive and sums to 100%. Effectiveness reflects each study’s own overall assessment across all the metrics it reported, rather than classification accuracy alone.

## Data Availability

No new primary data were created in this study. The data supporting the reported results, including the data-charting form and the list of excluded full-text reports with reasons for exclusion, are available in the Supplementary Materials.

## Supplementary Materials

The following supporting information can be downloaded at https://www.mdpi.com/article/10.3390/jintelligence14090191/s1. References (Almanza-Conejo et al., 2024; Alves-Oliveira et al., 2019; Choi & Cho, 2020; Correia et al., 2019; David et al., 2021; Duville et al., 2021; Flynn et al., 2020; Kurian, 2023; Lai et al., 2024; Law et al., 2021; Lekkas et al., 2023; D. Li, 2024; Loftness et al., 2024; McStay, 2020; Mirheidari et al., 2024; Neves et al., 2021; Pasupa & Seneewong Na Ayutthaya, 2019; Revathi & Manikandan, 2023; Saliba et al., 2023; Thangavel et al., 2019; Tiwari & Agarwal, 2021; Van Royen et al., 2022; Vural et al., 2024; Wairagkar et al., 2022; Zhu et al., 2024) are cited in supplementary file.

## Abbreviations

The following abbreviations are used in this manuscript:

AER	Automatic Emotion Recognition
AI	Artificial Intelligence
CNN	Convolutional Neural Network
PCC	Population, Concept, and Context
PRISMA-ScR	Preferred Reporting Items for Systematic Reviews and Meta-Analyses extension for Scoping Reviews
SVM	Support Vector Machine
VR	Virtual Reality
XAI	Explainable Artificial Intelligence
