# Supplementary material for: Decoding Social Cues: A Scoping Review of AI-Supported Emotional Competencies and Their Impact on School-Aged Populations
Source: J Intell. 2026 Sep 1;14(9):191. doi: 10.3390/jintelligence14090191 (PMC13608411; doi:10.3390/jintelligence14090191)
Supplement: Supplementary file 1 [file jintelligence-14-00191-s001.zip › jintelligence-4239799-supplementary.pdf]

Supplementary Table S1. Boolean search strings by database

Note. *The seven search strings combine the same four thematic blocks (AI, emotion, technology, and child/adolescent population), adapted to the syntax of each platform.*

Web of Science

```
(ALL=("artificial intelligence" OR "machine learning" OR "deep learning" OR "AI Techniques" OR "AI")AND ALL=("emotion*" OR "affective computing" OR "simulation" OR "empathy")AND ALL=("serious games" OR "technology" OR "virtual reality" OR "VR" OR "biofeedback" OR "neurofeedback" OR "avatar")AND ALL=("child*" OR "preschool" OR "teenager*" OR "adolescent*" OR "kid*"))
```

Scopus

```
TITLE-ABS-KEY("artificial intelligence" OR "machine learning" OR "deep learning" OR "AI techniques" OR AI)AND TITLE-ABS-KEY(emotion* OR "affective computing" OR simulation OR empathy)AND TITLE-ABS-KEY("serious games" OR technology OR "virtual reality" OR VR OR biofeedback OR neurofeedback OR avatar)AND TITLE-ABS-KEY(child* OR preschool OR teenager* OR adolescent* OR kid*)
```

PubMed

```
("artificial intelligence"[tiab] OR "machine learning"[tiab] OR "deep learning"[tiab] OR "AI techniques"[tiab] OR AI[tiab])
AND (emotion*[tiab] OR "affective computing"[tiab] OR simulation[tiab] OR empathy[tiab])
AND ("serious games"[tiab] OR technology[tiab] OR "virtual reality"[tiab] OR VR[tiab] OR biofeedback[tiab] OR neurofeedback[tiab] OR avatar[tiab])
AND (child*[tiab] OR preschool[tiab] OR teenager*[tiab] OR adolescent*[tiab] OR kid*[tiab])
```

APA (PsycNet)

```
1  (artificial intelligence or machine learning or deep learning or AI techniques or AI).af.
2  (emotion* or affective computing or simulation or empathy).af.
3  (serious games or technology or virtual reality or VR or biofeedback or neurofeedback or avatar).af.
4  (child* or preschool or teenager* or adolescent* or kid*).af.
5  1 and 2 and 3 and 4
```

ERIC

```
TX( "artificial intelligence" OR "machine learning" OR "deep learning" OR "AI techniques" OR AI )AND TX( emotion* OR "affective computing" OR simulation OR empathy )AND TX( "serious games" OR technology OR "virtual reality" OR VR OR biofeedback OR neurofeedback OR avatar )AND TX( child* OR preschool OR teenager* OR adolescent* OR kid*)
```

IEEE Xplore

("All Metadata":("artificial intelligence" OR "machine learning" OR "deep learning" OR "AI techniques" OR AI))AND ("All Metadata":(emotion* OR "affective computing" OR simulation OR empathy))AND ("All Metadata":("serious games" OR technology OR "virtual reality" OR VR OR biofeedback OR neurofeedback OR avatar))AND ("All Metadata":(child* OR preschool OR teenager* OR adolescent* OR kid*))

ACM Digital Library

("artificial intelligence" OR "machine learning" OR "deep learning" OR "AI techniques" OR AI) AND (emotion* OR "affective computing" OR simulation OR empathy)
AND ("serious games" OR technology OR "virtual reality" OR VR OR biofeedback OR neurofeedback OR avatar) AND (child* OR preschool OR teenager* OR adolescent* OR kid*)

Supplementary Table S2

Full-text articles excluded after eligibility assessment and primary reasons for exclusion

Note. This table is for direct use as supplementary material and aligns with PRISMA item 16b. Each study is assigned only one primary reason for exclusion.

No.	Study	Title	Primary reason for exclusion
1	Almanza-Conejo et al. (2024)	Emotion Recognition in Gaming Dataset to Reduce Artifacts in the Self-Assessed Labeling Using Semi-Supervised Clustering	Wrong outcome
2	Kurian (2023)	AI's empathy gap: The risks of conversational Artificial Intelligence for young children's well-being and key ethical considerations for early childhood education and care	Not an empirical study
3	David et al. (2021)	Effectiveness of the REThink therapeutic online video game in promoting mental health in children and adolescents	Did not use artificial intelligence
4	Van Royen et al. (2022)	Think Twice to be Nice? A User Experience Study on a Reflective<br>Interface to Reduce Cyber Harassment on Social Networking Sites	Wrong outcome
5	Lai et al. (2024)	The application of artificial intelligence technology in education influences Chinese adolescents' emotional perception	Wrong outcome

No.	Study	Title	Primary reason for exclusion
6	Saliba et al. (2023)	PISTACHIo (PreemptIon of diSrupTive behAvior in CHIldren): real-time monitoring of sleep and behavior of children 3–7 years old receiving parent–child interaction therapy augment with artificial intelligence — the study protocol, pilot study	Study protocol
7	Neves et al. (2021)	Associations between vocal emotion recognition and socio-emotional adjustment in children	Did not use artificial intelligence
8	McStay (2020)	Emotional AI and EdTech: Serving the Public Good?	Not an empirical study
9	Loftness et al. (2024)	The ChAMP App: A Scalable mHealth Technology for Detecting Digital Phenotypes of Early Childhood Mental Health	Wrong population
10	D. Li (2024)	An interactive teaching evaluation system for preschool education in universities based on machine learning algorithm	Wrong outcome
11	Wairagkar et al. (2022)	Emotive Response to a Hybrid-Face Robot and Translation to Consumer Social Robots	Wrong outcome
12	Tiwari & Agarwal (2021)	A Shrewd Artificial Neural Network-Based Hybrid Model for Pervasive Stress Detection of Students Using Galvanic Skin Response and Electrocardiogram Signal	Wrong outcome

No.	Study	Title	Primary reason for exclusion
13	Vural et al. (2024)	Stress recognition from facial images in children during physiotherapy with serious games	Wrong population
14	Law et al. (2021)	Automatic voice emotion recognition of child-parent conversation in natural setting	Wrong outcome
15	Correia et al. (2019)	Resting-state connectivity reveals a role for sensorimotor systems in vocal emotional processing in children	Wrong outcome
16	Choi & Cho (2020)	Bayesian networks reinforcement learning: Controlling group emotion from sensory stimuli	Wrong population
17	Zhu et al. (2024)	Defining Standard Values for FaceReader Facial Expression software output	Wrong outcome
18	Mirheidari et al. (2024)	Automatic detection of expressed emotion from Five-minute speech samples: challenges and opportunities	Wrong population
19	Pasupa & Seneewong Na Ayutthaya (2019)	Thai sentiment analysis with deep learning techniques: A comparative study based on word embedding, POS -tag, and sentic features	Wrong outcome

No.	Study	Title	Primary reason for exclusion
20	Revathi & Manikandan (2023)	A Smart and Secured Approach for Children's Health monitoring using machine learning techniques enhancing data privacy	Wrong outcome
21	Lekkas et al. (2023)	A machine learning investigation into the temporal dynamics of physical activity-mediated emotional regulation in adolescents with anorexia nervosa and healthy controls	Wrong population
22	Duville et al. (2021)	Mexican Emotional Speech Database Based on Semantic, Frequency, Familiarity, Concreteness, and Cultural Shaping of Affective Prosody	Wrong outcome
23	Flynn et al. (2020)	Assessing the Effectiveness of Automated Emotion Recognition in Adults and Children for Clinical Investigation	Wrong population
24	Alves-Oliveira et al. (2019)	Empathic Robot for Group Learning: A Field Study	Wrong outcome
25	Thangavel et al. (2019)	Deep Learning Based Emotion Analysis Approach for Strengthening Teaching-Learning Process in Schools of Tribal Regions	Wrong outcome

Twenty-five full-text reports were excluded following eligibility assessment. The excluded reports and the primary reason for exclusion for each report are presented in Supplementary Table S2.

Supplementary Table S3

Note. Twenty-five full-text reports were excluded following eligibility assessment.

Eligibility criteria: C1 = empirical study with a quantitative design; C2 = published 2019–2024 in a peer-reviewed journal indexed in WoS, Scopus, PubMed, PsycNet, ERIC, IEEE Xplore, or ACM Digital Library; C3 = participants aged 17 years or younger; C4 = employed AI-based technology addressing emotional competencies (affective computing, emotion recognition, physiological regulation, or immersive environments); C5 = published in English.

Additional exclusion criteria applied alongside eligibility screening: research not related to education; non-empirical studies (reviews, theoretical articles, editorials); studies on clinical or non-normotypical populations.

No.	Study	Eligibility criterion not met (C1–C5)	Additional exclusion criterion	Justification
1	Almanza-Conejo et al. (2024)	C3 + C4		Technical/algorithmic study using an existing EEG gaming dataset (FEEL); participant age not verifiable as ≤17; outcome is classifier performance, not an emotional competency.
2	Kurian (2023)	C1	Non-empirical study	Conceptual colloquium/ethical discussion; no empirical sample or quantitative design.
3	David et al. (2021)	C4		Meets C1, C2, C3, C5 (*N* = 31, ages 10–16, Internet Interventions, English). Sole failure: no AI-based technology used.
4	Van Royen et al. (2022)	C4		Uses machine learning, but the outcome measured is user experience/intention to harass, not an emotional competency.

No.	Study	Eligibility criterion not met (C1–C5)	Additional exclusion criterion	Justification
5	Lai et al. (2024)	C4		Meets C1, C2 (WoS/Scopus-indexed), C3, C5. The AI used is general AIEd, not one of the defined emotional-AI categories.
6	Saliba et al. (2023)	C1		Study protocol/pilot with no outcome results yet.
7	Neves et al. (2021)	C4		Human perceptual/behavioral task; no AI-based technology involved.
8	McStay (2020)	C1	Non-empirical study	Critical/conceptual essay; no empirical data collected.
9	Loftness et al. (2024)	C3	Clinical/non-normotypical population	Sample of 50 children (5–8y) with and without anxiety/depression disorders — a clinical mental-health sample, not typically developing. Also arguably not education-related.
10	D. Li (2024)	C3 + C4		Participants are university preschool-education students/teachers, not children; outcome is teaching-quality evaluation, not a child emotional competency.
11	Wairagkar et al. (2022)	C3 + C4		Participants are adults (19–29y); outcome is robot design/validation, not a child's emotional competency.
12	Tiwari & Agarwal (2021)	C3 + C4		Participants are university students (adults); outcome is stress-classifier accuracy, not an emotional competency.
13	Vural et al. (2024)	C3	Clinical/non-normotypical population	AKTIVES dataset: children with obstetric brachial plexus injury, dyslexia, and intellectual disabilities (plus typical controls) in a physiotherapy context — clinical/non-normotypical sample and not education-related.
14	Law et al. (2021)	C4		Outcome is automatic voice-emotion-recognition algorithm accuracy, not the child's emotional competency.

No.	Study	Eligibility criterion not met (C1–C5)	Additional exclusion criterion	Justification
15	Correia et al. (2019)	C4		fMRI resting-state connectivity study; no AI-based technology used.
16	Choi & Cho (2020)	C3 + C4		Data collected from both a kindergarten and a senior welfare center (mixed/non-child setting); outcome is ambient stimuli-control system performance, not individual emotional competency.
17	Zhu et al. (2024)	C3 + C4		Human evaluators (*n* = 496 survey respondents) are adults; outcome is technical validation of facial-expression software output.
18	Mirheidari et al. (2024)	C3 + C4		Subjects analyzed are caregivers/mothers speaking about their children, not the children themselves; outcome is algorithm accuracy for detecting expressed emotion in speech.
19	Pasupa & Seneewong Na Ayutthaya (2019)	C3 + C4		Analyzes text from Thai children's tales (no real child participants); outcome is sentiment-classification accuracy.
20	Revathi & Manikandan (2023)	C4		Correct child population (ages 0–10); outcome is IoT health-monitoring/data-privacy performance, not an emotional competency.
21	Lekkas et al. (2023)	C3	Clinical/non-normotypical population	Sample of 75 adolescents: 44 with anorexia nervosa and 31 healthy controls — a clinical eating-disorder sample; not education-related.
22	Duville et al. (2021)	C3 + C4		Corpus includes both adult (mean age ~22–24) and child voice actors; outcome is validation of a speech database, not an emotional competency.

No.	Study	Eligibility criterion not met (C1–C5)	Additional exclusion criterion	Justification
23	Flynn et al. (2020)	C3		Explicit mixed sample of adults and children; does use AI-based emotion-recognition technology (meets C4).
24	Alves-Oliveira et al. (2019)	C4		Correct child population; uses an AI-based empathic robot (meets C4); outcome is collaborative learning gains, not an emotional competency.
25	Thangavel et al. (2019)	C4		The study evaluates improvement in the teaching–learning process rather than students' emotional competencies

Supplementary Table S4

Eligibility criteria: C1 = empirical study with a quantitative design; C2 = published 2019–2024 in a peer-reviewed journal indexed in WoS, Scopus, PubMed, PsycNet, ERIC, IEEE Xplore, or ACM Digital Library; C3 = participants aged 17 years or younger; C4 = employed AI-based technology addressing emotional competencies (affective computing, emotion recognition, physiological regulation, or immersive environments); C5 = published in English.

Full-text articles included

No.	Study	C1	C2	C3	C4	C5
1	Ali et al. (2022)	✓	✓	✓	✓	✓
2	Calvo-Barajas et al. (2021)	✓	✓	✓	✓	✓
3	Han et al. (2021)	✓	✓	✓	✓	✓
4	Kory-Westlund & Breazeal (2019)	✓	✓	✓	✓	✓
5	Kalaiyarasi et al. (2024)	✓	✓	✓	✓	✓
6	Kim et al. (2021)	✓	✓	✓	✓	✓
7	X. Li (2022)	✓	✓	✓	✓	✓
8	S. Li et al. (2019)	✓	✓	✓	✓	✓
9	Xiao et al. (2024)	✓	✓	✓	✓	✓

No.	Study	C1	C2	C3	C4	C5
10	Lopez-Larrosa et al. (2023)	✓	✓	✓	✓	✓
11	Qayyum et al. (2023)	✓	✓	✓	✓	✓
12	Rathod et al. (2022)	✓	✓	✓	✓	✓
13	Savchenko et al. (2022)	✓	✓	✓	✓	✓
14	Veiga Simão et al. (2021)	✓	✓	✓	✓	✓
15	Xie et al. (2022)	✓	✓	✓	✓	✓
16	Xu et al. (2024)	✓	✓	✓	✓	✓
17	Yan (2022)	✓	✓	✓	✓	✓
18	Yun et al. (2020)	✓	✓	✓	✓	✓
